# Synthesis of Chromonylthiazolidines and Their Cytotoxicity to Human Cancer Cell Lines

**DOI:** 10.3390/molecules20011151

**Published:** 2015-01-12

**Authors:** Hoang Le Tuan Anh, Nguyen Thi Cuc, Bui Huu Tai, Pham Hai Yen, Nguyen Xuan Nhiem, Do Thi Thao, Nguyen Hoai Nam, Chau Van Minh, Phan Van Kiem, Young Ho Kim

**Affiliations:** 1 Institute of Marine Biochemistry, Vietnam Academy of Science and Technology, 18 Hoang Quoc Viet, Caugiay, Hanoi 10000, Vietnam; E-Mails: anh_792002@yahoo.com (H.L.T.A.); cuc_k51a@yahoo.com (N.T.C.); bhtaiich@gmail.com (B.H.T.); yeninpc@yahoo.com (P.H.Y.); nxnhiem@yahoo.com (N.X.N.); namnguyenhoai@imbc.vast.vn (N.H.N.); cvminh@vast.ac.vn (C.V.M.); phankiem@yahoo.com (P.V.K.); 2 College of Pharmacy, Chungnam National University, Daejeon 305-764, Korea; 3 Institute of Biotechnology, Vietnam Academy of Science and Technology, 18 Hoang Quoc Viet, Caugiay, Hanoi 10000, Vietnam; E-Mail: thaodo74@yahoo.com

**Keywords:** anti-cancer agent, chromonylthiazolidine, *Paeonia lactiflora*, paeonol, selective cytotoxicity, semi-synthesis

## Abstract

Nine new chromonylthiazolidine derivatives were successfully semi-synthesized from paeonol. All of the compounds, including starting materials, the intermediate compound and products, were evaluated for their cytotoxic effects toward eight human cancer cell lines. The synthesized chromonylthiazolidines displayed weak cytotoxic effects against the tested cancer cell lines, but selective cytotoxic effects were observed. Compounds **3a** and **3b** showed the most selective cytotoxic effects against human epidermoid carcinoma (IC_50_ 44.1 ± 3.6 μg/mL) and breast cancer (IC_50_ 32.8 ± 1.4 μg/mL) cell lines, respectively. The results suggest that chromoylthiazolidines are potential low-cost, and selective anticancer agents.

## 1. Introduction

Cancer is one of the most serious global health threats. It is characterized by explosive proliferation of cells. To date, over 100 types of cancer have been classified on the basis of the initially affected cells. Annually, more than 11 million people are diagnosed with cancer and it is estimated that up to 16 million new cases will occur annually by 2020 [1]. Cancer has various causes, including genetic factors, lifestyle, chemicals, and radiation exposure. Cancer patients can be saved if the cancer is detected at an early stage and effective treatment is provided. Cancer treatment depends on the type of cancer and its stage, but it usually comprises a combination of therapies such as surgery, radiation, chemotherapy, and gene therapy. Among them, chemotherapy remains an important option for treatment of cancer [2]. Many chemotherapeutic agents, such as 5-fluorouracil, cisplatin, paclitaxel and docetaxel, have been approved and are currently used to treat cancer [3]. The main goal of anti-cancer agents is to induce apoptosis of cancer cells. However, in some cases, the dose of anticancer drug needed to kill cancer cells is toxic to normal cells and hence leads to many undesired side effects. Moreover, decreased drug activity or development of drug resistance during long-term treatment is believed to cause failure in over 90% of patients with metastatic cancer [4,5]. Therefore, discovery of novel anticancer agents with high efficacy, selectivity, low costs and minimal side effects, and new approaches to cancer treatment, is an exciting prospect.

Thiazolidine rings, notably 1,4-thiazolidinedione (TZD), have been used as a scaffold to develop novel anticancer agents with a broad spectrum of cytotoxicity toward various human cancer cells [6,7]. Recently, TZD derivatives were emphasized as a new dawn in cancer chemotherapy [8,9]. Various preclinical and clinical studies strongly suggest that the anticancer activity of TZDs is mediated in part by PPAR-γ-dependent or -independent pathways. Several new TZDs, such as efatutazone, netoglitazone, rosiglitazone and troglitazone, are being explored in terms of the mechanism underlying their anticancer activity [8]. Having researched anticancer agents containing a thiazolidine core motif, we herein report the synthesis of nine new chromonylthiazolidines and evaluation of their cytotoxicity against several human cancer cell lines.

## 2. Results and Discussion

In the past decade, thiazolidines were emphasized as a new class of compounds with a wide range of pharmacological activities, such as antihyperglycemic, euglycemic, anti-inflammatory, antimalarial, antioxidant, antitumor, cytotoxic, antimicrobial, and antiproliferative effects [10,11,12]. The literature also indicates that thiazolidines act as PPAR agonists and inhibit various important enzymes, such as cyclin-dependent kinases (CDKs), glycogen synthase kinase (GSK), aldose reductase and bacterial arylamine N-acetyltransferase (NAT) [8,10]. Due to their broad biological spectrum, thiazolidine molecules have attracted the attention of medicinal chemists and a number of synthesis strategies have been developed.

Chromone derivatives are abundant in all parts of plants and vegetables, such as flowers, fruits, seeds and bark [13,14]. They show low mammalian toxicity and are present in large amounts in food [15]. Many naturally occurring molecules contain chromone in their structures, especially flavonoids, which exhibit a wide range of biological activities [14,16]. Therefore, we hypothesized that chromonylthiazolidines might have a broad spectrum of pharmacological activities as well as selective cytotoxicity toward cancer cell lines. We thus aimed to design and optimize chromonylthiazolidines as a potential anti-cancer candidate by anti-proliferative effects against several human cancer cell lines.

**Scheme 1:**
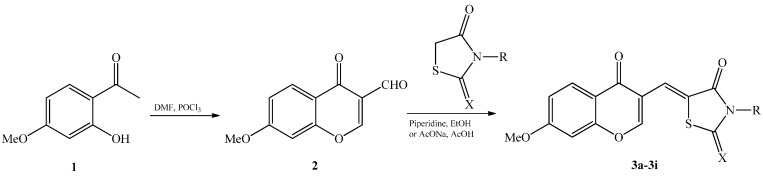
Synthesis of the chromonylthiazolidines.

In our strategy, chromonylthiazolidines **3a**–**i** were synthesized by a Knoevenagel condensation reaction between 3-formyl-7-methoxychromone (**2**) and different thiazolidine derivatives (Scheme 1). First, 3-formyl-7-methoxychromone (**2**) was semi-synthesized from the starting material paeonol (**1**), which was isolated in abundance from *Paeonia lactiflora* (see Experimental Section). Through the Vilsmeier–Haack reaction, compound **2** was easily obtained by treatment of paeonol with dimethylformamide and phosphorous oxychloride [17]. Next, compound **2** was reacted with appropriate thiazolidine derivatives in the presence of piperidine as a base catalyst to give compounds **3a**–**i**. However, the reaction yields of compounds **3g**–**i** were low, so anhydrous sodium acetate and glacial acetic acid were used as the catalyst and reaction medium, respectively, to improve reaction yields. Finally, chromonylthiazolidines **3a**–**i** were obtained with reaction yields ranging from 38% to 72% (see Experimental Section, Table 1). The structures of the synthesized compounds were elucidated on the basis of ^1^H-NMR, ^13^C-NMR and ESI-MS spectra. All spectral data were in accordance with assumed structures (see Experimental Section). Chromonylthiazolidines **3a**–**i**, paeonol (**1**), and the intermediate compound **2** were evaluated for their effects on the viability of various human cancer cell lines: HepG2 (hepatocellular carcinoma), HL-60 (acute promyeloid leukemia), KB (epidermoid carcinoma), LLC (Lewis lung carcinoma), LNCaP (hormone dependent prostate cancer), LU-1 (human lung cancer), MCF7 (breast cancer) and SW480 (colon adenocarcinoma). Briefly, cancer cells were cultured with various concentrations of synthetic compounds (0.8–100 μg/mL) for 3 days. Then, the cells were incubated with MTT solution for 4 h. Formation of a violet precipitate, formazan, was monitored in DMSO at a wavelength of 540 nm using a microplate reader. The cytotoxicity of compounds is expressed as IC_50_ values. Ellipticine is an efficient anticancer drug. It functions by number of mechanisms such as participating in cell cycle arrest, initiation of apoptosis, DNA-damaging [18,19]. Thus, in our experiments, ellipticine was used as a positive control. Its IC_50_ values for HepG2, HL-60, KB, LLC, LNCaP, LU-1, MCF-7 and SW480 cells were 1.45 ± 0.08, 0.56 ± 0.04, 0.43 ± 0.05, 0.98 ± 0.04, 0.86 ± 0.06, 1.29 ± 0.11, 0.49 ± 0.04 and 0.62 ± 0.05 μg/mL, respectively. Our results indicate that the synthesized chromonylthiazolidines exhibit weak cytotoxic effects toward the eight tested human cancer cell lines (Table 2). However, the results also suggest selective cytotoxic effects of chromonylthiazolidines toward cancer cells. Notably, compound **3a** showed weak cytotoxic effects against KB and LU-1 cells (IC_50_ 44.1 ± 3.6 and 52.9 ± 3.4 μg/mL, respectively), but it did not show potent cytotoxic effects against the other cancer cell lines. Similarly, compound **3b** exhibited stronger cytotoxicity only against MCF-7 cells (IC_50_ 32.8 ± 1.4) among the eight selected cancer cell lines. In addition, the chromonylthiazolidine-2,4-dione derivatives **3a**–**c** displayed cytotoxic effects stronger than those of the chromonylthiazolidine-2-thione-4-one derivatives **3d**–**i**. Compound **3c** exhibited modest cytotoxic effects toward all tested cancer cell lines. Meanwhile, compounds **3e** and **3g** showed significant selective cytotoxic effects against half of the tested cancer cell lines (HepG2, HL-60, LLC and SW480). All of the synthesized chromonylthiazolidines showed weak cytotoxicity against HL-60 and SW480 cells: IC_50_ values were 74.6 ± 4.6 to 94.8 ± 6.8 μg/mL and 71.4 ± 3.6 to 92.5 ± 4.12 μg/mL, respectively.

**Table 1:** Synthesized chromonylthiazolidines **3a**–**i**. 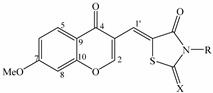

Compounds	X	R	Yield (%)
**3a**	O	H	72
**3b**	O	CH_3_	47
**3c**	O	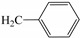	38
**3d**	S	NH_2_	66
**3e**	S	CH_3_	60
**3f**	S	H_2_C-CH=CH_2_	72
**3g**	S	H	55
**3h**	S	H_2_C-COOH	50
**3i**	S	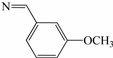	66

**Table 2:** Cytotoxicity of compounds **1**, **2**, and **3a**–**i** toward various human cancer cell lines.

Comp.	IC_50_ (μg/mL)							
	HepG2	HL-60	KB	LLC	LNCaP	LU-1	MCF7	SW480
**1**	>100	>100	>100	>100	>100	>100	>100	>100
**2**	>100	>100	>100	>100	>100	>100	>100	>100
**3a**	>100	82.2 ± 4.5	44.1 ± 3.6	87.4 ± 6.3	77.4 ± 5.8	52.9 ± 3.4	66.0 ± 2.7	71.4 ± 3.6
**3b**	86.3 ± 6.4	75.3 ± 3.9	84.6 ± 4.2	>100	81.6 ± 6.3	>100	32.8 ± 1.4	90.1 ± 4.8
**3c**	78.4 ± 5.8	92.3 ± 5.2	74.1 ± 5.1	90.1 ± 7.7	84.2 ± 4.1	65.5 ± 4.1	52.7 ± 3.6	85.4 ± 7.4
**3d**	94.5 ± 6.3	74.6 ± 4.6	90.2 ± 2.4	>100	92.4 ± 5.7	59.0 ± 4.3	88.4 ± 7.4	84.3 ± 6.2
**3e**	81.4 ± 7.4	94.8 ± 6.8	>100	90.2 ± 4.9	>100	>100	>100	87.6 ± 4.8
**3f**	78.4 ± 3.4	87.6 ± 6.7	>100	>100	91.6 ± 7.6	>100	65.9 ± 4.7	81.3 ± 5.6
**3g**	88.4 ± 6.7	84.6 ± 5.4	>100	90.8 ± 5.2	>100	>100	>100	90.8 ± 6.4
**3h**	91.3 ± 8.2	94.2 ± 7.1	88.4 ± 3.5	87.4 ± 6.4	81.9 ± 4.3	85.6 ± 5.6	>100	85.7 ± 3.2
**3i**	>100	84.3 ± 4.2	90.2 ± 2.7	92.8 ± 6.4	85.1 ± 6.1	>100	80.9 ± 6.9	92.5 ± 4.1
Ellipticine	1.45 ± 0.08	0.56 ± 0.04	0.43 ± 0.05	0.98 ± 0.04	0.86 ± 0.06	1.29 ± 0.11	0.49 ± 0.04	0.62 ± 0.05

## 3. Experimental Section

### 3.1. General Experimental Procedures

Melting points were determined on an Electrothermal 9100 melting point apparatus. NMR spectroscopy was recorded on AVANCE 500 MHz (Bruker, Karlsruhe, Germany) instrument. Electrospray ionization mass spectrometry (ESI-MS) was measured by Agilent 1100 Series LC/MSD Trap SL (Agilent Technologies, Waldbronn, Germany). HR-ESI-MS spectra were recorded on MicroQ-TOP III mass spectrometer (Bruker Daltonics, Bremen, Germany). 3-Methyl-2,4-thiazolidinedione (used for the synthesis of **3b**) and *N*-(3-methoxybenzyliden)-3-amino-2-thione-4-thiazolidinone (using for the synthesis of **3i**) were synthetic compounds. They were easily obtained from reactions between 2,4-thiazolidinedione/3-amino-2-thione-4-thiazolidinone and iodomethane/3-methoxybenzaldehyde, respectively [20,21]. Other chemical reagents were purchased from Sigma-Aldrich (St. Louis, MO, USA).

### 3.2. Isolation of Paeonol (**1**)

Dried and powdered of *Paeonia lactiflora* leaves (3 kg) were extracted with chloroform (20 L × 3 times) at room temperature for 3 times. After removal of solvent, a chloroform extract (130 g) was obtained. This crude extract was separated on a silica gel chromatography column eluted with a gradient solvent system of *n*-hexane/acetone (1/0, 50/1, 10/1, 5/1, and 1/1, each 3 L stepwise) to give five fractions C1‒C5. Fraction C3 (67 g) was recrystallized several times in methanol to obtain paeonol (**1**, 11.4 g, 0.38% w/w of dried material) [22]. ESI-MS: *m/z* 167.0 [M+H]^+^; ^1^H-NMR (500 MHz, CDCl_3_) δ (ppm): 12.73 (1H, s, OH), 7.63 (1H, d, *J* = 8.0 Hz, H-Ar), 6.44 (1H, dd, *J* = 8.0, 2.0 Hz, H-Ar), 6.42 (1H, d, *J* = 2.0 Hz, H-Ar), 3.83 (3H, s, OCH_3_), 2.55 (3H, s, COCH_3_). ^13^C-NMR (125 MHz, CDCl_3_) δ (ppm): 202.5, 166.1, 165.3, 132.3, 113.9, 107.6, 100.9, 55.5, 26.2.

### 3.3. Synthesis of 3-Formyl-7-methoxychromone (**2**)

A solution of paeonol (**1**, 3.32 g, 0.02 mol) in *N*,*N'*-dimethylformamide (DMF, 10 mL) was added to a 100 mL round-bottomed flask and stirred in an ice bath (0–5 °C). Next, phosphorus oxychloride (POCl_3_, 4 mL) was slowly added. After that, the reaction mixture was warmed and kept at room temperature for 30 min. The reaction solution was poured into iced water. The reaction product was filtered, washed with distilled water, and re-crystalized in a solvent system of *n*-hexane/ethyl acetate to yield 3-formyl-7-methoxychromone (**2**, 3.08 g, 76%) [23]. ESI-MS: *m/z* 205.2 [M+H]^+^, 227.0 [M+Na]^+^; ^1^H-NMR (500 MHz, CDCl_3_) δ_H_ (ppm): 10.38 (1H, d, *J* = 4.0 Hz, CHO), 8.48 (1H, brs, H-2), 8.21 (1H, d, *J* = 9.0 Hz, H-5), 7.06 (1H, d, *J* = 9.0 Hz, H-6), 6.92 (1H, s, H-8), 3.93 (3H, s, OCH_3_).

### 3.4. Synthesis of Chromonylthiazolidines **3a**‒**i**

#### 3.4.1. Procedure 1

Compound 2 (100 mg, 0.5 mmol) and 2,4-thiazolidinedione or 2-thione-4-thiazolidinone derivatives (0.5 mmol) were dissolved in ethanol (5 mL). Piperidine (100 μL) was added as a catalyst and the reaction mixture was stirred at room temperature for 5 h. Then, the reaction mixture was poured into iced water to yield a precipitate. The product was filtered, washed with distilled water and recrystallized from the indicated solvents to yield compounds **3a**‒**f**.

*5-((7-Methoxy-4-oxo-4H-chromen-3-yl)methylene)thiazolidine-2,4-dione* (**3a**): From EtOH/DMF (1:1); Mp. 183–184 °C; HR-ESI-MS *m/z*: 304.0261 [M+H]^+^ (calcd [C_14_H_10_O_5_NS]^+^ for 304.0274), 326.0085 [M+Na]^+^ (calcd [C_14_H_9_O_5_NSNa]^+^ for 326.0094); ESI-MS *m/z*: 304.0 [M+H]^+^; ^1^H-NMR (500 MHz, DMSO-*d*_6_) δ_H_ ppm: 12.38 (1H, s, NH), 8.72 (1H, s, H-2), 8.02 (1H, d, *J* = 8.5 Hz, H-5); 7.57 (1H, s, H-1'), 7.19 (1H, s, H-8), 7.12 (1H, d, *J* = 8.5 Hz, H-6), 3.91 (3H, s, 7-OCH_3_); ^13^C-NMR (125 MHz, DMSO-*d*_6_) δ_C_ ppm: 173.8, 169.0, 167.3, 164.3, 160.2, 126.9, 124.8, 124.4, 117.7, 116.6, 115.4, 100.9, 56.2.

*5-((7-Methoxy-4-oxo-4H-chromen-3-yl)methylene)-3-methyl-thiazolidine-2,4-dione* (**3b**): From EtOH/DMF (1:1); Mp. 186–187 °C; HR-ESI-MS *m/z*: 340.0246 [M+Na]^+^ (calcd [C_15_H_11_O_5_NSNa]^+^ for 340.0250); ESI-MS: *m/z* 318.1 [M+H]^+^; ^1^H-NMR (500 MHz, DMSO-*d*_6_) δ_H_ (ppm): 8.83 (1H, s, H-2), 8.03 (1H, d, *J* = 9.0 Hz, H-5), 7.69 (1H, s, H-1'), 7.23 (1H, d, *J* = 2.0 Hz, H-8), 7.14 (1H, dd, *J* = 9.0, 2.0, H-6), 3.92 (3H, s, 7-OCH_3_), 3.08 (3H, s, NCH_3_).

*3-Benzyl-5-((7-methoxy-4-oxo-4H-chromen-3-yl)methylene)thiazolidine-2,4-dione* (**3c**): From EtOH/toluene (1:1); Mp. 211–212 °C; HR-ESI-MS *m/z*: 416.0551 [M+Na]^+^ (calcd [C_21_H_15_O_5_NSNa]^+^ for 416.0563); ESI-MS: *m/z* 394.0 [M+H]^+^; ^1^H-NMR (500 MHz, DMSO-*d*_6_) δ_H_ (ppm): 8.85 (1H, s, H-2), 8.03 (1H, d, *J* = 9.0 Hz, H-5), 7.72 (1H, s, H-1'), 7.33–7.28 (5H, m, H-Phenyl), 7.23 (1H, d, *J* = 2.0 Hz, H-8), 7.14 (1H, dd, *J* = 9.0, 2.0 Hz, H-6), 4.81 (2H, s, NCH_2_), 3.92 (3H, s, 7-OCH_3_).

*3-Amino-5-((7-methoxy-4-oxo-4H-chromen-3-yl)methylene)-2-thioxothiazolidin-4-one* (**3d**): From EtOH; Mp. 219–220 °C; HR-ESI-MS *m/z*: 335.0140 [M+H]^+^ (calcd [C_14_H_11_O_4_N_2_S_2_]^+^ for 335.0155), 356.9967 [M+Na]^+^ (calcd [C_14_H_10_O_4_N_2_S_2_Na]^+^ for 356.9974); ESI-MS: *m/z* 335.0 [M+H]^+^; ^1^H-NMR (500 MHz, DMSO-*d*_6_) δ_H_ (ppm): 8.95 (1H, s, H-2), 8.03 (1H, d, *J* = 8.5 Hz, H-5), 7.57 (1H, s, H-1'), 7.23 (1H, s, H-8), 7.14 (1H, d, *J* = 8.5 Hz, H-6), 3.92 (3H, s, 7-OCH_3_); ^13^C-NMR (125 MHz, DMSO-*d*_6_) δ_C_ ppm: 191.1, 174.0, 164.5, 163.9, 163.1, 157.2, 127.1, 126.9, 121.5, 117.9, 116.5, 115.6, 101.3, 56.2.

*5-((7-Methoxy-4-oxo-4H-chromen-3-yl)methylene)-3-methyl-2-thioxothiazolidin-4-one* (**3e**): From EtOAc; Mp. 197–198 °C; HR-ESI-MS *m/z*: 356.0014 [M+Na]^+^ (calcd [C_15_H_11_O_4_NS_2_Na]^+^ for 356.0022); ESI-MS: *m/z* 334.0 [M+H]^+^; ^1^H-NMR (500 MHz, DMSO-*d*_6_) δ_H_ (ppm): 8.95 (1H, s, H-2), 8.04 (1H, d, *J* = 9.0 Hz, H-5), 7.55 (1H, s, H-1'), 7.24 (1H, s, H-8), 7.15 (1H, d, *J* = 9.0 Hz, H-6), 3.92 (1H, s, 7-OCH_3_), 3.38 (3H, s, NCH_3_).

*3-Allyl-5-((7-methoxy-4-oxo-4H-chromen-3-yl)methylene)-2-thioxothiazolidin-4-one* (**3f**): From EtOAc; Mp. 202–203 °C; HR-ESI-MS *m/z*: 382.0169 [M+Na]^+^ (calcd [C_17_H_13_O_4_NS_2_Na]^+^ for 382.0178); ESI-MS: *m/z* 360.0 [M+H]^+^; ^1^H-NMR (500 MHz, DMSO-*d*_6_) δ_H_ (ppm): 8.95 (1H, s, H-2), 8.03 (1H, d, *J* = 9.0 Hz, H-5), 7.54 (1H, s, H-1'), 7.23 (1H, d, *J* = 2.5 Hz, H-8), 7.14 (1H, dd, *J* = 9.0; 2.5 Hz, H-6), 5.85 (1H, m, H-Vinyl), 5.17 (1H, dd, *J* = 10.5; 1.0 Hz, H-Vinyl), 5.10 (1H, dd, *J* = 17.5; 1.0 Hz, H-Vinyl), 4.62 (2H, d, *J* = 5.0 Hz, NCH_2_), 3.91 (3H, s, 7-OCH_3_); ^13^C-NMR (125 MHz, DMSO-*d*_6_) δ_C_ (ppm): 196.4, 174.10, 166.8, 164.5, 163.0, 157.2, 130.3, 127.0, 126.8, 123.5, 117.8, 117.5, 116.5, 115.6, 101.1, 56.2, 45.8.

#### 3.4.2. Procedure 2

A solution of compound **2** (100 mg, 0.5 mmol), 2-thione-4-thiazolidinone derivatives (0.5 mmol), and sodium acetate (50 mg) in glacial acetic acid (5 mL) was added to a 50 mL round-bottomed flask. The reaction mixture was refluxed for 2 h and then poured into ice water to yield a precipitate. The product was filtered, washed with distilled water and recrystallized from ethanol (unless otherwise indicated) to yield compounds **3g**‒**i**.

*5-((7-Methoxy-4-oxo-4H-chromen-3-yl)methylene)-2-thioxothiazolidin-4-one* (**3g**): From EtOH; Mp. 192–193 °C; HR-ESI-MS *m/z*: 320.0031 [M+H]^+^ (calcd [C_14_H_10_O_4_NS_2_]^+^ for 320.0046), 341.9857 [M+Na]^+^ (calcd [C_14_H_9_O_4_NS_2_Na]^+^ for 341.9865); ESI-MS: *m/z* 320.0 [M+H]^+^, 318.0 [M−H]^−^; ^1^H-NMR (500 MHz, DMSO-*d*_6_) δ_H_ (ppm): 8.86 (1H, s, H-2), 8.02 (1H, d, *J* = 9.0 Hz, H-5), 7.37 (1H, s, H-1'), 7.22 (1H, d, *J* = 2.5 Hz, H-8), 7.13 (1H, dd, *J* = 9.0, 2.5 Hz, H-6), 3.91 (3H, s, 7-OCH_3_). ^13^C-NMR (125 MHz, DMSO-*d*_6_) δ_C_ (ppm): 198.6, 174.1, 164.5, 163.9, 162.2, 157.2, 127.0, 117.7, 116.6, 115.6, 101.0, 56.3.

*2-(5-((7-Methoxy-4-oxo-4H-chromen-3-yl)methylene)-4-oxo-2-thioxothiazolidin-3-yl)acetic acid* (**3h**): From EtOH; Mp. 223–224 °C; HR-ESI-MS *m/z*: 378.0101 [M+H]^+^ (calcd [C_16_H_12_O_6_NS_2_]^+^ for 378.0101), 399.9912 [M+Na]^+^ (calcd [C_16_H_11_O_6_NS_2_Na]^+^ for 399.9920); ESI-MS: *m/z* 378.0 [M+H]^+^, 375.9 [M−H]^−^; ^1^H-NMR (500 MHz, DMSO-*d*_6_) δ_H_ ppm: 8.97 (1H, s, H-2), 8.04 (1H, d, *J* = 8.5 Hz, H-5), 7.58 (1H, s, H-1'), 7.24 (1H, d, *J* = 2.5 Hz, H-8), 7.14 (1H, dd, *J* = 8.5, 2.5 Hz, H-6), 4.17 (2H, s, NCH_2_), 3.92 (3H, s, 7-OCH_3_); ^13^C-NMR (125 MHz, DMSO-*d*_6_) δ_C_ ppm: 196.7, 174.2, 167.3, 166.7, 164.6, 163.4, 157.3, 127.0, 127.0, 123.2, 117.7, 116.5, 115.7, 101.0, 56.3, 44.9.

*5-((7-Methoxy-4-oxo-4H-chromen-3-yl)methylene)-3-((3-methoxybenzylidene)amino)-2-thioxothiazol-idin-4-one* (**3i**): From EtOH/toluene (1:1); Mp. 234–235 °C; HR-ESI-MS *m/z*: 475.0381 [M+Na]^+^ (calcd [C_22_H_16_O_5_N_2_S_2_Na]^+^ for 475.0393); ESI-MS: *m/z* 453.0 [M+H]^+^; ^1^H-NMR (500 MHz, DMSO-*d*_6_) δ_H_ ppm: 9.02 (1H, s, N=CH), 8.88 (1H, s, H-2), 8.07 (1H, d, *J* = 8.5 Hz, H-5), 7.61 (1H, s, H-1'), 7.49 (3H, overlapped, H-Ar), 7.27 (1H, d, *J* = 2.5 Hz, H-8), 7.24 (1H, m, H-Ar), 7.17 (1H, dd, *J* = 8.5, 2.5 Hz, H-6), 3.93 (3H, s, 7-OCH_3_), 3.84 (3H, s, Ar-OCH_3_).

### 3.5. Cell Culture and Cell Viability Assay

Eight human cancer cell lines including HepG2 (hepatocellular carcinoma), HL-60 (acute promyeloid leukemia), KB (epidermoid carcinoma), LLC (Lewis lung carcinoma), LNCaP (hormone dependent prostate cancer), LU-1 (human lung cancer), MCF7 (breast cancer), and SW480 (colon adenocarcinoma) were used for cytotoxic evaluation. Cells were obtained from the American Type Culture Collection (Manassas, VA, USA) and cultured in DMEM or RPMI-1640 containing 2 mM L-glutamine, 1.5 g/L sodium bicarbonate, 4.5 g/L glucose, 10 mM HEPES, 1.0 mM sodium pyruvate and supplement with fetal bovine serum (FBS) 10%. The MCF7 medium was further added with 0.01 mg/mL bovine insulin while LNCaP medium was supplied with 10 nM of testosterone. The cells were incubated at 37 °C in a humidified atmosphere (95% air and 5% CO_2_).

The inhibitory effects of compounds on the growth of human cancer cell lines were determined by measuring metabolic activity using a 3-[4,5-dimethylthiazol-2-yl]-2,5-diphenyltetrazolium bromide (MTT) assay as previous described [24]. Briefly, human cancer cell lines (1 × 10^5^ cells/mL) were treated for 3 days with series concentrations 0.8, 4.0, 20.0, 50.0, and 100.0 µg/mL of the compounds dissolved in DMSO (100 mg/mL for stock solution, and a final concentration of DMSO in all experiments was 0.1%, v/v). After incubation, 0.1 mg (50 µL of a 2 mg/mL solution) MTT solution was added to each well and the cells were then incubated at 37 °C for 4 h. The culture medium was then carefully aspirated and dimethylsulfoxide (150 µL) was added to each well to dissolve the formazan crystals. The plates were read immediately at 540 nm on a microplate reader (TECAN GENIOUS). All the experiments were performed three times and the mean absorbance values were calculated. The results are expressed as the percentage of inhibition that produced a reduction in the absorbance by the treatment of the compounds compared to the untreated controls. A dose-response curve was generated and the inhibitory concentration of 50% (IC_50_) was determined for each compound as well as each cell line. Ellipticine was used as a positive control.

### 3.6. Statistical Analysis

All data were expressed as mean ± S.D. of at least three independent experiments performed in triplicates. Statistical significance is indicated by one-way ANOVA followed by Dunnett’s multiple comparison test using GraphPad Prism 6 program (GraphPad Software Inc., San Diego, CA, USA), *p* < 0.05.

## 4. Conclusions

In summary, the semi-synthesis of chromonylthiazolidines from paeonol, which was isolated in abundance from *P. lactiflora*, was inexpensive and gave good yields. The assumed structures of the synthesized compounds agreed with their ^1^H-NMR, ^13^C-NMR and HR-ESI-MS spectra data. All compounds were evaluated in term of their cytotoxic effects against eight human cancer cell lines: HepG2, HL-60, KB, LLC, LNCaP, LU-1, MCF-7 and SW480 cell lines. The synthesized chromonylthiazolidines displayed weak cytotoxic effects against the tested cancer cell lines, but selective cytotoxic effects were observed, suggesting that chromonylthiazolidines could be low-cost, selective anticancer agents.
